# Development and validation of nomograms for predicting survival in differentiated thyroid cancer patients with or without radioiodine therapy

**DOI:** 10.3389/fonc.2023.1054594

**Published:** 2023-03-09

**Authors:** Bingyu Ran, Jian Gong, Jingjie Shang, Feng Wei, Hao Xu

**Affiliations:** Department of Nuclear Medicine, The First Affiliated Hospital of Jinan University, Guangzhou, Guangdong, China

**Keywords:** thyroid cancer, radioiodine therapy, nomogram, overall survival, SEER

## Abstract

**Objective:**

This study aimed to establish and validate the nomograms for predicting overall survival (OS) probabilities in differentiated thyroid cancer (DTC) patients who received and did not receive radioiodine therapy (RAI), respectively.

**Methods:**

In this study, 11, 099 patients diagnosed with DTC in the Surveillance, Epidemiology, and End Results (SEER) database from 2004 to 2016 were selected. Whether they have RAI, they are divided into RAI (n=6427) and non-RAI (n=4672) groups. They were randomly assigned to either a training cohort (RAI: n=4498, non-RAI: n=3263) or a validation cohort (RAI: n=1929, non-RAI: n=1399) using R software to divide the patients in a 7-to-3 ratio randomly. Variables were selected using a backward stepwise method in a Cox regression model to determine the independent prognostic factors, which were then utilized to build two nomograms to predict the 5-, 8-, and 10-year OS probabilities in DTC patients with or without RAI. The concordance index (C‐index), the area under the time-dependent receiver operating characteristics curve (AUC), the net reclassification improvement (NRI), the integrated discrimination improvement (IDI), calibration plotting, and decision-curve analysis (DCA) were used to evaluate the performance of our models.

**Results:**

The multivariate analyses demonstrated that birth of the year, race, histological type, tumor size, grade, TNM stage, lymph node dissections, surgery, and chemotherapy were risk factors for OS. Compared to the AJCC stage, the C‐index (RAI: training group: 0.911 vs. 0.810, validation group: 0.873 vs. 0.761; non-RAI: training group: 0.903 vs. 0.846, validation group: 0.892 vs. 0.808). The AUC values for the training cohort (RAI: 0.940, 0.933, and 0.942; non-RAI: 0.891, 0.884, and 0.852 for the 5-, 8-, and 10-year OS, respectively) and validation cohort (RAI: 0.855, 0.825, and 0.900, non-RAI: 0.867, 0.896, and 0.899), and the calibration plots of both two models all exhibited better performance. Additionally, the NRI and IDI further showed that they exhibited good 5-, 8-, and 10-year net benefits.

**Conclusion:**

We have established the prediction models of DTC patients with or without RAI respectively through various variables. The nomogram may be more targeted to guide clinical decisions in the future.

## 1 Introduction

Thyroid cancer (TC) is the most prevalent cancer of the endocrine system (1), and its main pathological types are: DTC -including papillary, follicular, and Hürthle cells- medullary thyroid carcinoma (MTC), and undifferentiated thyroid carcinoma (ATC) (2). Papillary thyroid carcinoma (PTC) is the most common histological type of TC (3). The prevalence of TC has multiplied in recent decades, accounting for approximately 1-3% of human cancers (1), and is predicted to reach the fourth most common cancer by 2030 (4). There is broad agreement that DTC demonstrates relatively indolent clinical behavior. Significant improvements in the diagnosis and treatment of DTC have provided an excellent prognosis. In general, overall survival rates for TC are high, especially in patients with DTC, with 10-year survival rates ranging from 80 to 95 percent (5, 6). However, some studies in recent years have found an increase in mortality among patients with advanced-stage PTC (7, 8). This suggests a renewed focus on the prognosis and survival of thyroid cancer patients.

Thyroidectomy, RAI, and thyroid-stimulating hormone suppression are the primary treatment options for patients with DTC. Selective RAI therapy plays a vital role in removing potentially residual DTC and the treatment of distant metastases (9). To determine if patients need more aggressive treatment, the 2015 American Thyroid Association (ATA) guidelines developed a risk stratification system to identify patients at low, intermediate, or high-risk of thyroid cancer recurrence or death. They strongly recommend RAI for patients with a high risk of recurrence stratum. RAI may be considered for patients in the intermediate-risk stratum and is not routinely recommended after thyroidectomy for ATA low-risk DTC patients and patients with single lesion/multifocal microcarcinoma (≤1 cm in diameter) (10). However, consideration of specific features of the individual patient that could modulate recurrence risk, disease follow-up implications, and patient preferences are relevant to RAI decision-making (11, 12). So not all of the patients with DTC will be treated with RAI, and the indication and modalities of radioiodine treatment are shifting from a standardized practice to a tailored approach.

At present, there are no predictions for the survival of individual DTC patients based on RAI. In this study, based on the SEER database, we analyzed the mortality rates of DTC patients treated with or without radioiodine at 5, 8, or 10 years, which may accurately provide valuable individualized prognostic information. At the same time, these two nomograms can serve as a new reference to assist clinicians in selecting personalized RAI for DTC patients.

## 2 Methods

### 2.1 Source of data

The data we selected were obtained from the Surveillance, Epidemiology, and End Results (SEER) database (covering 18 registries) using the latest SEER*Stat version 8.3.5 (https://seer.cancer.gov/). The SEER Program is an authoritative data source of clinical information on cancer incidence and survival. It contains statistical information on cancer for approximately 35% of the U.S. population. All data from the SEER database was free, and we signed the SEER Research Data Agreement for this study to access SEER information using the username 19803-Nov2020. This study was approved by the Institutional Research Committee of the First Affiliated Hospital of Jinan University.

### 2.2 Patient selection

**Figure 1:**
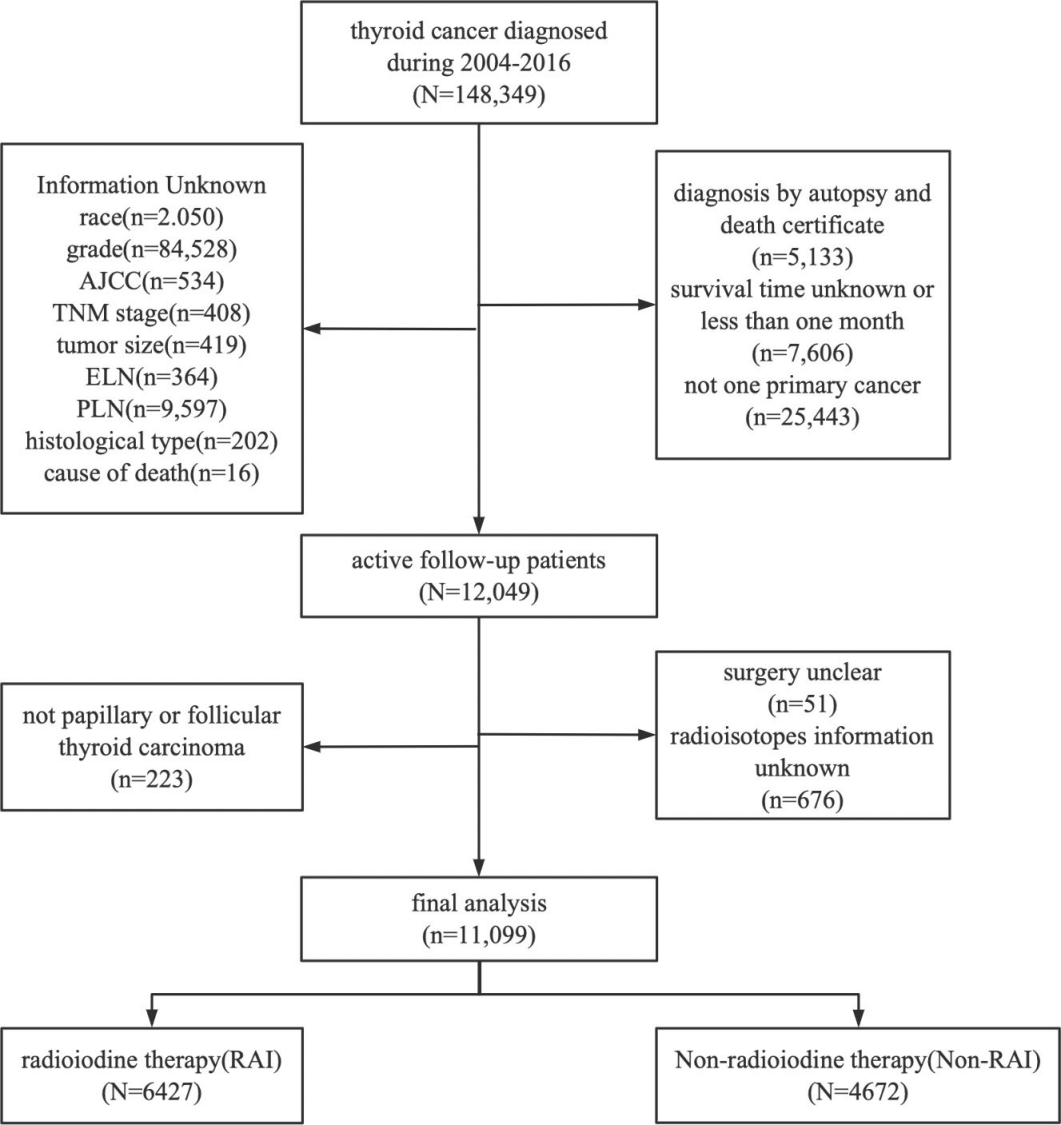
Flowchart of patient selection. Detailed selection of DTC patients in 2004–2016 from SEER database.

The location codes collected from the SEER database were TC C73.9, diagnosed with TC from 2004 to 2016. There were 148, 349 qualified patients identified in the SEER database, of which 11, 099 were available after applying a strict screening process (**Figure 1**). Then according to whether they have RAI, they are divided into RAI (n=6427) and non-RAI (n=4672) groups. For the construction and validation of the nomograms, we randomly distributed 70% of the patients to the training cohort (RAI: n=4498, non-RAI: n=3263) and 30% to the validation cohort (RAI: n=1929, non-RAI: n=1399). The following main variables were evaluated: the age of diagnosis, sex, race, histological type, tumor size, grade, AJCC stage, TNM stage, lymph node dissections (LND), the number of examined lymph nodes (ELN), the number of positive lymph nodes (PLN), the ratio of PLN and ELN (LNR), surgery, chemotherapy, follow-up time and all-cause death.

### 2.3 Nomogram development and statistical analyses

In the RAI group, the eleven pathological and clinical characteristics of the age of diagnosis, race, tumor size, grade, T\M stage, and chemotherapy were used to conduct the analyses, while in non-RAI, age of diagnosis, race, tumor size, grade, TNM stage, PLN, and chemotherapy were used for analysis. The backward stepwise selection method in a Cox regression model was applied to the training cohort to select variables. Using these identified prognostic factors, we then established two nomograms that predicted the 5-, 8-, and 10-year OS probabilities in DTC patients according to whether they have RAI, respectively.

After establishing the nomograms, internal and external validation is performed separately in the training and validation cohorts. We first used the C‐index and the receiver operating characteristic curve (ROC). The new model’s ability to discriminate is evaluated by assessing the AUC. Calibration plots were used to determine the agreement between the actual results and the predicted probabilities. Then to complement the accuracy and comprehensiveness of the comparison, two relatively new metrics (NRI and IDI) were used. Finally, the clinical value of the prediction model was determined by quantifying the net benefit at different threshold probabilities using DCA curves. Descriptive statistics were calculated and presented as the frequency (percentage) for categorical variables, the mean ± standard for continuous variables of normal distribution, and the median (interquartile range) for continuous variables of skewness distribution. All statistical analyses were conducted using R software (version 3.5.1; https://www.r-project.org). A two‐sided probability value of P ≤ 0.05 was deemed to indicate statistical significance.

## 3 Results

### 3.1 Patient characteristics

We evaluated a total of 11, 099 DTC patients from 2004 to 2016. These in groups RAI and non-RAI were divided by R software, respectively, using the popular random split-sample method (with a split ratio of 7: 3) into 4498 and 3263 in the training cohort and into 1929 and 1399 in the validation cohort. **Tables 1**, **2** summarized the demographic and tumor characteristics of the patients.

**Table 1:** Patient characteristics for RAI in the study.

Variables	All patients	Train set	Validation set	
**n**	6427	4498	1929	
**Age of diagnosis**	43.83 ± 14.87	43.95 ± 14.89	43.54 ± 14.83	P=0.310
**Sex**				P=0.893
**Male**	1652 (25.70%)	1154 (25.66%)	498 (25.82%)	
**Female**	4775 (74.30%)	3344 (74.34%)	1431 (74.18%)	
**Race**				p=0.733
**White**	5200 (80.99%)	3628 (80.66%)	1572 (81.49%)	
**Black**	243 (3.81%)	173 (3.85%)	70 (3.63%)	
**Other**	984 (15.21%)	697 (15.50%)	287 (14.88%)	
**Histological type**				p=0.012
**Papillary**	6068 (94.41%)	4268 (94.89%)	1800 (93.31%)	
**Follicular**	359 (5.59%)	230 (5.11%)	129 (6.69%)	
**Tumor size**	24.14 ± 17.75	24.10 ± 18.13	24.26 ± 16.82	p=0.743
**Grade**				p=0.484
**I**	5020 (78.11%)	3525 (78.37%)	1495 (77.50%)	
**II**	1124 (17.49%)	783 (17.41%)	341 (17.68%)	
**III**	254 (3.95%)	168 (3.73%)	86 (4.46%)	
**IV**	29 (0.45%)	22 (0.49%)	7 (0.36%)	
**AJCC**			1	p=0.887
**I**	4075 (63.40%)	2852 (63.41%)	1223 (63.40%)	
**II**	372 (5.79%)	259 (5.76%)	113 (5.86%)	
**III**	1149 (17.88%)	797 (17.72%)	352 (18.25%)	
**IV**	831 (12.93%)	590 (13.12%)	241 (12.49%)	
**T**				p=0.830
**1**	2516 (39.15%)	1774 (39.44%)	742 (38.47%)	
**2**	1402 (21.81%)	984 (21.88%)	418 (21.67%)	
**3**	2133 (33.19%)	1481 (32.93%)	652 (33.80%)	
**4**	376 (5.85%)	259 (5.76%)	117 (6.07%)	
**N**				p=0.471
**0**	2745 (42.71%)	1908 (42.42%)	837 (43.39%)	
**1**	3682 (57.29%)	2590 (57.58%)	1092 (56.61%)	
**M**				p=0.481
**0**	6315 (98.26%)	4423 (98.33%)	1892 (98.08%)	
**1**	112 (1.74%)	75 (1.67%)	37 (1.92%)	
**Lymph node dissections**				p=0.106
**None or Biopsy**	138 (2.15%)	90 (2.00%)	48 (2.49%)	
**1–3 regional LN**	2571 (40.00%)	1771 (39.37%)	800 (41.47%)	
**≥ 4 regional LN**	3662 (56.98%)	2593 (57.65%)	1069 (55.42%)	
**Unknown**	56 (0.87%)	44 (0.98%)	12 (0.62%)	
**ELN**	11.89 ± 17.17	12.01 ± 17.27	11.62 ± 16.91	p=0.405
**PLN**	3.62 ± 6.39	3.65 ± 6.40	3.54 ± 6.38	p=0.536
**LNR**	0.29 ± 0.35	0.30 ± 0.35	0.29 ± 0.35	p=0.848
**Surgery**				p=0.231
**No surgery**	1 (0.02%)	0 (0.00%)	1 (0.05%)	
**Lobectomy**	146 (2.27%)	95 (2.11%)	51 (2.64%)	
**Subtotal or near-totalthyroidectomy**	115 (1.79%)	83 (1.85%)	32 (1.66%)	
**Thyroidectomy**	6165 (95.92%)	4320 (96.04%)	1845 (95.65%)	
**Chemotherapy**				p=0.681
**Yes**	23 (0.36%)	17 (0.38%)	6 (0.31%)	
**No/unknown**	6404 (99.64%)	4481 (99.262%)	1923 (99.69%)	
**status**				p=0.912
**Alive**	6246 (97.18%)	4372 (97.20%)	1874 (97.15%)	
**Death**	181 (2.82%)	126 (2.80%)	55 (2.85%)	

LND, lymph node dissections; ELN, the number of examined lymph nodes; PLN, the number of positive lymph nodes; LNR, the ratio of PLN and ELN.

**Table 2:** Patient characteristics for non-RAI in the study.

Variables	All patients	Train set	Validation set	
**n**	4672	3263	1399	
**Age of diagnosis**	45.83 ± 14.71	45.60 ± 14.79	46.35 ± 14.49	p=0.114
**Sex**				p=0.201
**Male**	907 (19.46%)	619 (18.97%)	288 (21.30%)	
**Female**	3755 (80.54%)	2644 (81.03%)	1111 (79.41%)	
**Race**				p=0.772
**White**	3938 (84.47%)	2758 (84.52%)	1180 (84.35%)	
**Black**	208 (4.46%)	149 (4.57%)	59 (4.22%)	
**Other**	516 (11.07%)	356 (10.91%)	160 (11.44%)	
**Histological type**				p=0.618
**Papillary**	4441 (95.26%)	3105 (95.16%)	1336 (95.50%)	
**Follicular**	221 (4.74%)	158 (4.84%)	63 (4.50%)	
**Tumor size**	16.71 ± 19.18	16.93 ± 20.29	16.20 ± 16.28	p=0.234
**Grade**				p=0.298
**I**	3934 (84.38%)	2733 (83.76%)	1201 (85.85%)	
**II**	594 (12.74%)	435 (13.33%)	159 (11.37%)	
**III**	100 (2.15%)	70 (2.15%)	30 (2.14%)	
**IV**	34 (0.73%)	25 (0.77%)	9 (0.64%)	
**AJCC**				p=0.963
**I**	3748 (80.39%)	2626 (80.48%)	1122 (80.20%)	
**II**	215 (4.61%)	147 (4.51%)	68 (4.86%)	
**III**	691 (14.82%)	302 (9.26%)	129 (9.22%)	
**IV**	144 (3.09%)	188 (5.76%)	80 (5.72%)	
**T**				p=0.466
**1**	3170 (68.00%)	2204 (67.55%)	966 (69.05%)	
**2**	657 (14.09%)	457 (14.01%)	200 (14.30%)	
**3**	691 (14.82%)	495 (15.17%)	196 (14.01%)	
**4**	144 (3.09%)	107 (3.28%)	37 (2.64%)	
**N**				p=0.739
**0**	3514 (75.38%)	2455 (75.24%)	1059 (75.70%)	
**1**	1148 (24.62%)	808 (24.76%)	340 (24.30%)	
**M**				p=0.553
**0**	4618 (99.06%)	3234 (99.11%)	1384 (98.93%)	
**1**	44 (0.94%)	29 (0.89%)	15 (1.07%)	
**Lymph node dissections**				p=0.028
**None or Biopsy**	150 (3.22%)	95 (2.91%)	55 (3.93%)	
**1–3 regional LN**	2696 (57.83%)	1873 (57.40%)	823 (58.83%)	
**≥ 4 regional LN**	1744 (37.41%)	1271 (38.95%)	503 (35.95%)	
**Unknown**	42 (0.90%)	24 (0.74%)	18 (1.29%)	
**ELN**	6.40 ± 11.28	6.28 ± 10.61	6.69 ± 12.70	p=0.297
**PLN**	1.27 ± 3.88	1.22 ± 3.58	1.40 ± 4.48	p=0.166
**LNR**	0.13 ± 0.28	0.13 ± 0.28	0.13 ± 0.28	p=0.601
**Surgery**				p=0.058
**No surgery**	4 (0.09%)	4 (0.12%)	0 (0.00%)	
**Lobectomy**	643 (13.79%)	450 (13.79%)	193 (13.80%)	
**Subtotal or near-total thyroidectomy**	126 (2.70%)	76 (2.33%)	50 (3.57%)	
**Thyroidectomy**	3869 (82.99%)	2733 (83.76%)	1156 (82.63%)	
**Chemotherapy**				p=0.217
**Yes**	8 (0.17%)	4 (0.12%)	4 (0.29%)	
**No/Unknown**	4654 (99.83%)	3259 (99.88%)	1395 (99.71%)	
**status**				p=0.616
**alive**	4521 (96.98%)	3167 (97.06%)	1354 (96.78%)	
**death**	141 (3.02%)	96 (2.94%)	45 (3.22%)	

LND, lymph node dissections; ELN, the number of examined lymph nodes; PLN, the number of positive lymph nodes; LNR, the ratio of PLN and ELN.

### 3.2 Variable screening

Age of diagnosis, sex, race, histological type, tumor size, grade, AJCC stage, TNM stage, LND, ELN, PLN, LNR, surgery, and chemotherapy was entered into multivariable Cox regression analysis. Finally, in the RAI group, we identified eleven independent prognostic factors: age of diagnosis (HR=1.086, p<0.001), Black (HR=1.994, p=0.070 vs. White), other (HR=0.472, p=0.006 vs. White), tumor size(HR=1.014, p<0.001), Grade II (HR=1.052, p=0.844 vs. Grade I), Grade III (HR=3.839, p=0.003 vs. Grade I), Grade IV (HR=3.849, p=0.001 vs. Grade I), T2 (HR=1.132, p=0.732 vs. T1), T3 (HR=2.039, p=0.015 vs T1), T4 (HR=3.559, p<0.001 vs. T1), M1 (HR=5.034, p<0.001 vs. M0), chemotherapy-yes (HR=0.278, p=0.004 vs. no chemotherapy).

In non-RAI, age of diagnosis (HR=1.078, p<0.001), Black (HR=1.323, p=0.449 vs. White), other (HR=0.611, p=0.088 vs. White), tumor size(HR=1.006, p=0.048), Grade II (HR=0.807, p=0.504 vs. Grade I), Grade III (HR=3.489, p<0.001 vs. Grade I), Grade IV (HR=6.952, p<0.001 vs. Grade I), T2 (HR=1.603, p=0.222 vs. T1), T3 (HR=1.342, p=0.398 vs T1), T4 (HR=3.636, p=0.001 vs. T1), N1 (HR=2.789, p<0.001 vs. N0), M1 (HR=2.027, p=0.052 vs. M0), PLN(HR=1.033, p=0.042), chemotherapy-yes (HR=0.079, p=0.001 vs. no chemotherapy). **Tables 3**, **4** list the multivariable Cox regression analysis results.

**Table 3:** Selected variables by multivariate Cox regression analysis (training cohort) OS for RAI.

Variables	Multivariate analysis		
	HR	95% CI	P‐value
**Age**	1.086	1.072-1.101	<0.001
Race			
**White**	Reference		
**Black**	1.994	0.945-4.204	0.070
**Other**	0.472	0.276-0.810	0.006
**Tumor size**	1.014	1.009-1.019	<0.001
Grade			
**I**	Reference		
**II**	1.052	0.642-1.781	0.844
**III**	3.839	1.567-9.401	0.003
**IV**	4.343	2.775-7.157	<0.001
T			
**1**	Reference		
**2**	1.132	0.558-2.295	0.732
**3**	2.039	1.145-3.631	0.015
**4**	3.559	1.847-6.856	<0.001
M			
**0**	Reference		
**1**	5.034	3.065-8.267	<0.001
Chemotherapy			
**Yes**	Reference		
**No/Unknown**	0.287	0.121-0.680	0.004

**Table 4:** Selected variables by multivariate Cox regression analysis (training cohort) OS for non-RAI.

Variables	Multivariate analysis		
	HR	95% CI	P‐value
**Age of diagnosis**	1.078	1.065-1.093	<0.001
Race			
**White**	Reference		
**Black**	1.323	0.641-2.279	0.449
**Other**	0.611	0.347-1.075	0.088
**Tumor size**	1.006	1.000-1.013	0.048
Grade			
**I**	Reference		
**II**	0.807	0.430-1.514	0.504
**III**	3.489	1.946-6.255	<0.001
**IV**	6.952	3.173-15.232	<0.001
T			
**1**	Reference		
**2**	1. 342	0.679-2.654	0.398
**3**	1. 603	0.751-3.420	0.222
**4**	3.636	1.744-7.581	0.001
N			
**0**	Reference		
**1**	2.789	1.672-4.650	<0.001
M			
**0**	Reference		
**1**	2.027	0.994-4.139	0.052
**PLN**	1.033	1.001-1.067	0.042
Chemotherapy			
**Yes**		Reference	
**No/Unknown**	0.079	0.017-0.374	0.001

PLN, the number of positive lymph nodes.

### 3.3 Nomogram establishment

**Figure 2:**
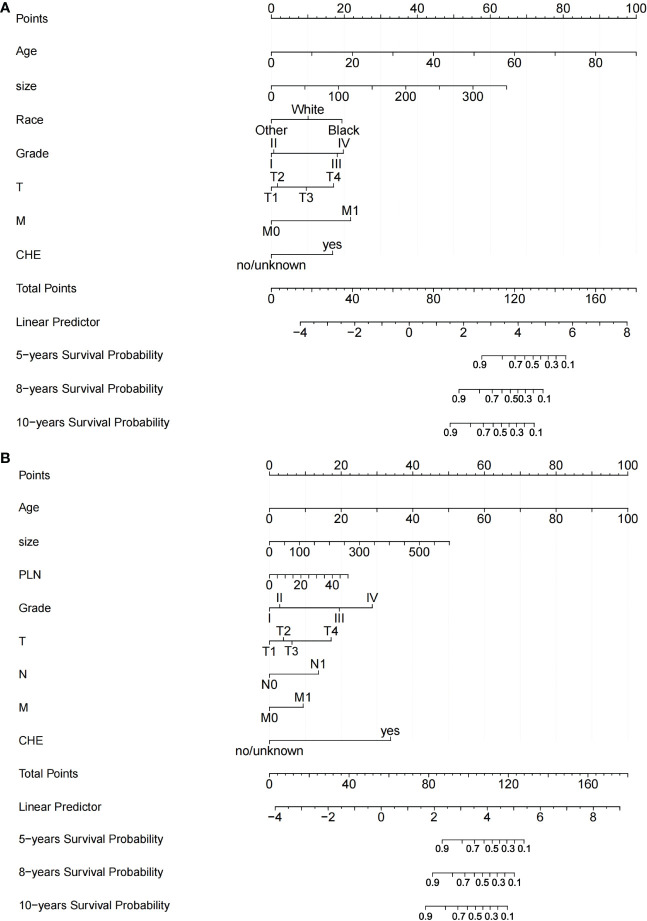
Nomograms for predicting OS of patients with DTC. **(A)** Nomogram for predicting 5‐, 8‐, and 10‐y OS of DTC patients with RAI; **(B)** Nomogram for predicting 5‐, 8‐, and 10‐y OS of DTC patients without RAI. TT: Thyroidectomy, PLN: the number of positive lymph nodes.

Two nomograms based on the selected prognostic factors from the training cohort were developed to predict OS probabilities in DTC patients with or without radioisotope, respectively, at 5, 8, and 10 years (**Figures 2A, B**). The nomogram for RAI demonstrated that age of diagnosis contributed the most to prognosis, followed by tumor size, M, Grade, race, T, and chemotherapy. In the OS nomogram for non-RAI, age of diagnosis was also the strongest influencing factor, followed by tumor size, chemotherapy, Grade, PLN, T, N, and M. A total score was obtained by adding the scores for each selected variable, and a vertical line is dropped down from the total-points row to estimate the OS for 5, 8, and 10 years (13).

### 3.4 Validation and calibration of the nomograms

**Figure 3:**
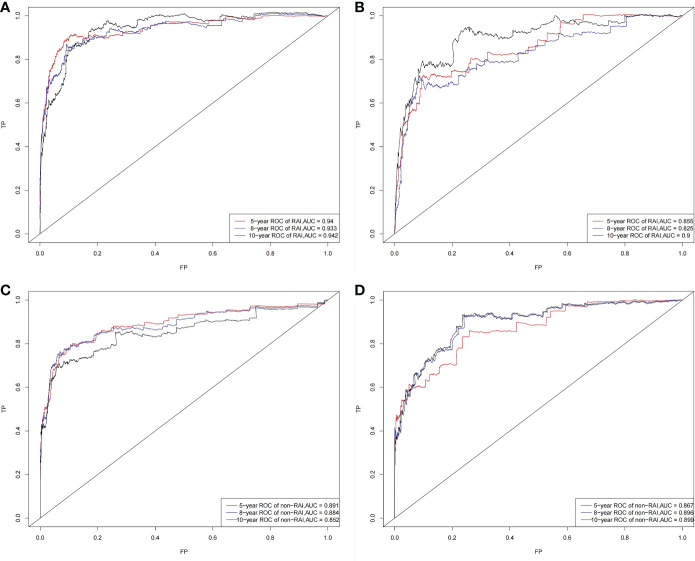
ROC curves. The ability of the model to be measured by the AUC. **(A)** came from the training set of RAI, and **(B)** came from the validation set of RAI; **(C)** came from the training set of non-RAI, and **(D)** came from the validation set of non-RAI.

The C‐index for the OS nomogram of RAI (training group=0.911, validation group=0.873) was higher than those based on the AJCC stage (training group=0.810, verification group=0.761). The AUC values for the training cohort (0.940, 0.933, and 0.942 for the 5‐, 8‐, and 10‐year OS, respectively) and validation cohort (0.855, 0.825, and 0.900) indicated the excellent discriminative ability of the model (**Figures 3A, B**). The C‐index for the OS nomogram of non-RAI was also higher for the nomogram than for the AJCC stage in both the training cohort (0.903 vs. 0.846) and the validation cohort (0.892 vs. 0.808). The AUC values for the training cohort (0.891, 0.884, and 0.852 for the 5‐, 8‐, and 10‐year OS, respectively) and validation cohort (0.867, 0.896, and 0.899) indicated the good identification ability of the model also for the OS nomogram of non-RAI (**Figures 3C, D**).

Analysis of accuracy for RAI showed that the NRI value for the 5-, 8- and 10-year follow-ups were 0.756 (95% confidence interval [CI] = 0.640-0.893), 0.596 (95% CI = 0.405-0.978), and 0.548 (95% CI = 0.369-0.821), respectively, in the training cohort, and 0.239 (95% CI = 0.031-0.445), 0.454 (95% CI = 0.139-0.670), and 0.597 (95% CI = 0.377-0.823) in the validation cohort. In addition, the IDI values for the 5-, 8-, and 10-year OS probabilities were 0.221, 0.304, and 0.304, respectively (p < 0.001), in the training cohort, 0.180, 0.233, and 0.179, respectively (p < 0.001), in the validation cohort.

In the non-RAI group, the NRI value for the 5-, 8- and 10-year follow-ups were 0.420 (95% CI = 0.257-0.609), 0.606 (95% CI = 0.361-0.788), and 0.579 (95% CI = 0.274-0.884), respectively, in the training cohort, and 0.384 (95% CI = 0.184-0.566), 0.522 (95% CI = 0.214-0.762), and 0.601 (95% CI = 0.249-0.951) in the validation cohort. The IDI values for the 5-, 8-, and 10-year OS probabilities were 0.226, 0.226, and 0.226, respectively (p < 0.001), in the training cohort, 0.194, 0.214, and 0.216, respectively (p < 0.001), in the validation cohort.

These values being more significant than zero indicate that our nomograms have a better potential for accurately predicting prognosis compared to the AJCC stage system.

**Figure 4:**
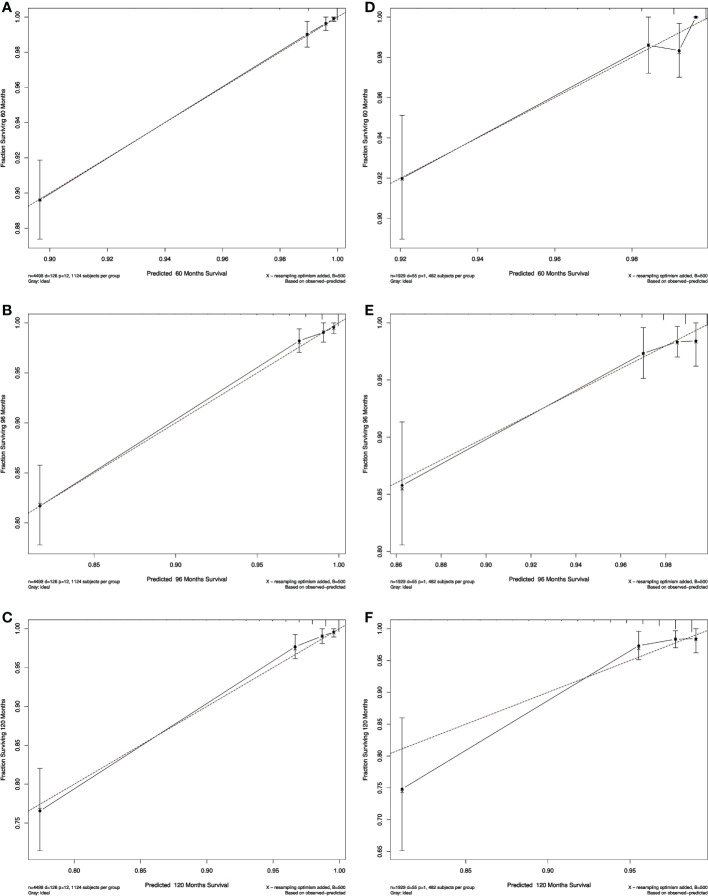
Calibration curves for RAI. Show the relationship between the predicted probabilities based on the nomogram and actual values of the train set **(A–C)** and validation set **(D–F)**.

**Figure 5:**
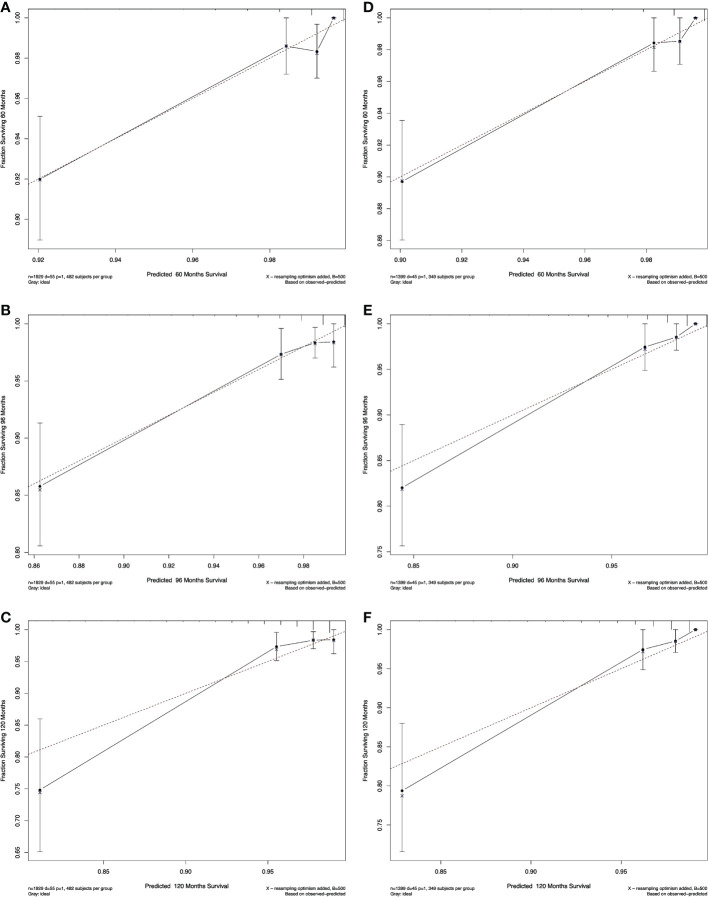
Calibration curves for non-RAI. Show the relationship between the predicted probabilities based on the nomogram and actual values of the train set **(A-C)** and validation set **(D-F)**.

The calibration plots of both nomograms showed excellent agreement between the actual observations and the predicted outcomes in both training and validation cohorts (**Figures 4**, **5**) for 5-, 8-, and 10-years OS.

**Figure 6:**
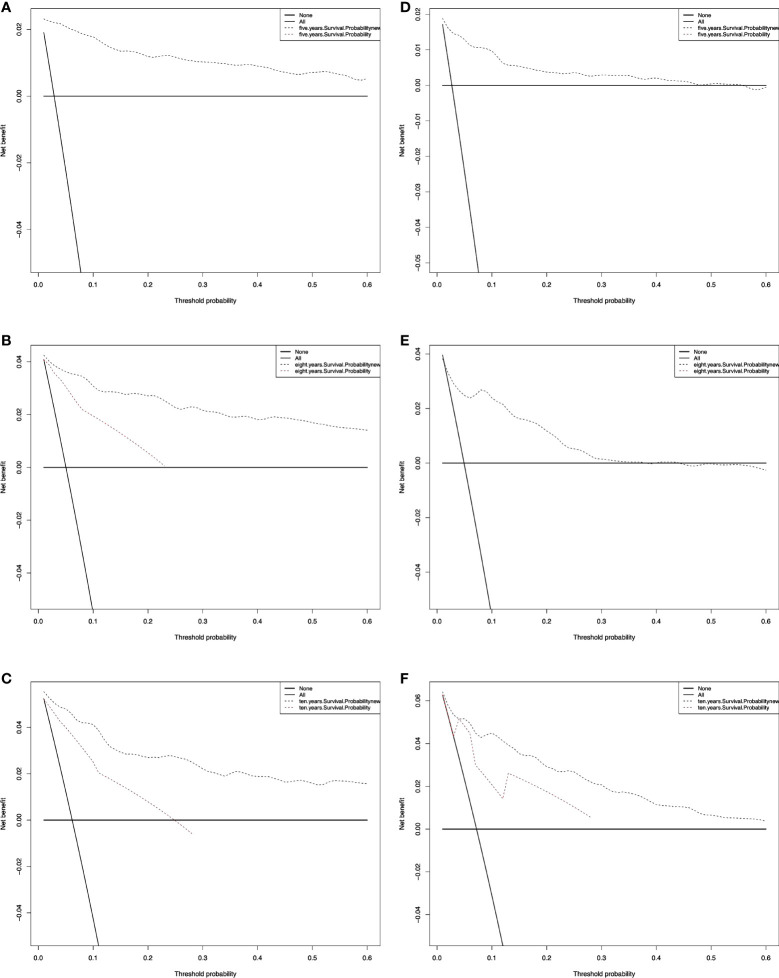
Decision curve analysis curves for RAI. Decision curve analysis of the training cohort **(A‐C)** and validation cohort **(D‐F)** for 5-, 8-, and 10-years cancer-specific survival probability.

**Figure 7:**
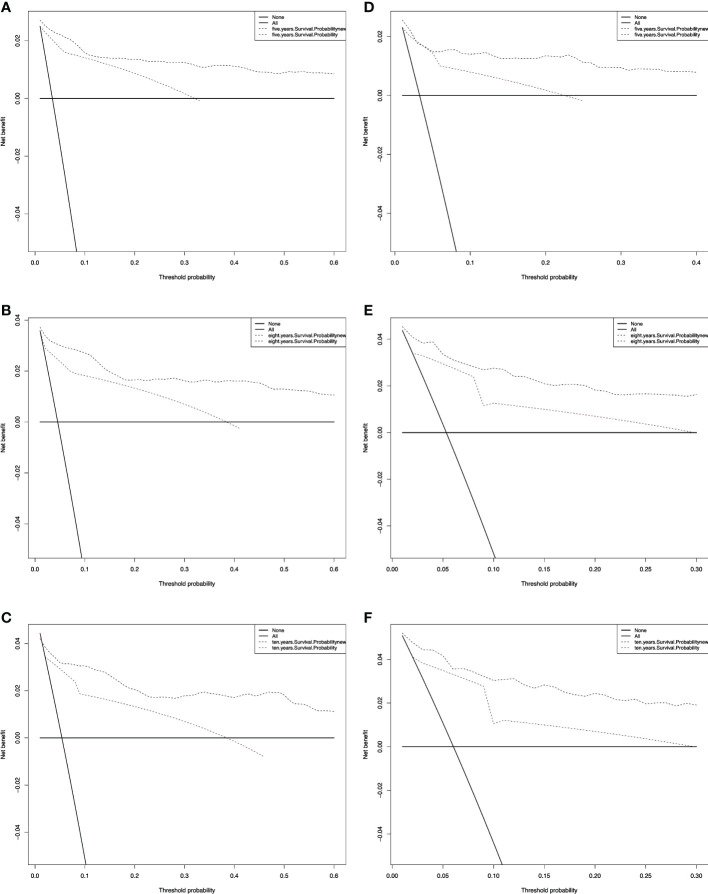
Decision curve analysis curves for non-RAI. Decision curve analysis of the training cohort **(A–C)** and validation cohort **(D–F)** for 5-, 8-, and 10-years cancer-specific survival probability.

We also performed a DCA, which showed that predicting the OS probabilities applying the two models yielded net benefits in the training and validation cohorts (**Figures 6**, **7**).

## 4 Discussion

Although the American Joint Committee on Cancer (AJCC) TNM staging system is beneficial in determining the prognosis of DTC patients (11), it ignores significant risk factors such as age, race, tissue type, and surgical status. With the advancement of precision medicine, the TNM staging system cannot provide comprehensive treatment guidance and prognosis prediction for individual patients. Also, the benefits of RAI application are currently controversial, especially in patients with low-risk DTC and microcarcinoma. Whether RAI can improve survival in these patients is a matter of intense scientific debate (14–18). As a result, although as a classic postoperative adjuvant therapy, RAI is not used for all patients with DTC. It is necessary to separately predict the survival probability of DTC patients based on RAI.

A nomogram, which translates complex regression equations into visual graphs, is a more widely used clinical tool that will facilitate patient counseling and personalized treatment. To date, no nomogram has been reported respectively for predicting the probability of survival in DTC patients with or without RAI. Therefore, we constructed two more comprehensive models to better predict DTC patients’ prognoses in this study. We analyzed the characteristics of DTC patients treated with or without RAI in the SEER database. Univariate and multifactorial analyses were performed to screen for independent risk factors for line graph modeling. Compared to the traditional AJCC staging system, the 7-variable scale based on age of diagnosis, race, tumor size, T, M, Grade, and chemotherapy in RAI and the 8-variable scale based on the age of diagnosis, tumor size, Grade, PLN, TNM, and chemotherapy in non-RAI were able to more accurate assessment and prediction of DTC patients in training and validation cohorts, further providing detailed forecast and follow-up plans for each patient.

The age presented higher coefficients and HR values in both univariate and multivariate analyses. Previous studies have shown that the adverse effect of age on outcome gradually increases with each decade, especially after the age of 40-45 year (19, 20). In the eighth edition of the AJCC TNM staging system, the threshold for age at diagnosis was changed from 45 years to 55 years. In our study, age, as an essential independent predictor, was not stratified, but a continuous age line was used to evaluate the prognosis of patients. Although DTC is 2-3 times more common in women than in men (21), in none of our studies was gender found to be an independent predictor of mortality from DTC either in patients receiving RAI or in those not receiving RAI (10). Consistent with the findings of Sunny Patel et al. Several other studies have highlighted that there is no significant difference in mortality between male and female patients (2, 22). Also in the AJCC cancer staging system, gender was not seen as an independent prognostic factor.

Nomogram enables us to find that chemotherapy is an independent risk factor for overall survival in patients with DTC. The 2015 ATA guidelines recommend systemic therapies (such as cytotoxic chemotherapy or kinase inhibitors) for loco-regional disease are considered after all surgical and radiation therapy options have been exhausted (10). In our study, all patients in the RAI group had undergone total thyroidectomy, consistent with the treatment regimen recommended by ATA guidelines. We speculate that these patients who require chemotherapy are in poor condition and are likely to have iodine-refractory thyroid cancer, so chemotherapy would be an independent risk factor for their prognosis. However, only one patient in the non-RAI group did not receive surgical treatment, and the rest underwent total thyroidectomy. Due to the lack of relevant information in the database, it is not possible to know why these patients were treated with chemotherapy rather than surgery and RAI. Therefore, in further studies, we will optimize history-taking and deeply explore the additional effects of chemotherapy on DTC patients.

The number of positive lymph nodes and the N-stage was independent risk factors for prognosis in DTC patients who did not receive RAI compared to patients who did. According to the guidelines, the vast majority of patients not recommended for RAI are low-risk patients with no or few lymph node metastases. For DTC patients receiving RAI, the level of risk should be reassessed based on the post-RAI status. The number of positive lymph nodes and stage N1 will be independent risk factors for patients at intermediate to high risk who should have received RAI but do not receive it. During the follow-up, in these patients, lymph node metastases should be closely monitored for real-time assessment of prognosis.

The strengths of this study include that it is a population-based study with a large sample size and is adjusted for the clinical demographic characteristics of the patients. These two nomograms can be used not only to predict the survival prognosis of DTC patients with and without RAI respectively, but also to assist in clinical and patient decision-making by assessing prognostic risk based on a patient’s current clinical profile when deciding whether to receive RAI. However, this study has several significant limitations. First, several potentially essential parameters and specific information related to prognosis were not available in the SEER database, making it less comprehensive, such as thyroid-stimulating hormone levels, thyroid hormone suppression treatment status, radioiodine treatment dose, etc. Second, our nomogram was internally validated but not externally validated, which may lead to overheating of the new model. Finally, the nomogram was constructed based on data from the SEER database and did not represent the global population. Therefore, we plan to conduct additional prospective studies in the future to test the denomination maps to compensate for these limitations.

## 5 Conclusions

To better determine the prognosis of DTC patients, this study establishes and validates, for the first time, 5-, 8-, and 10-year OS rates for DTC patients receiving or not receiving RAI, respectively, based on a large population-based cohort study. Compared with the AJCC staging system, the model showed superior predictive power and clinical usability, providing clinicians with more accurate reference information for personalized treatment and follow-up plans for DTC patients.

## Data availability statement

The datasets presented in this study can be found in online repositories. The names of the repository/repositories and accession number(s) can be found in the article/**Supplementary Material**.

## Author contributions

All authors contributed to the study conception and design. BR conceptualized and designed the study, collected and analyzed data, and wrote and edited the manuscript. BR, JS, and FW prepared the figures and tables. JG and HX confirm the authenticity of all the raw data. All authors contributed to the article and approved the submitted version.

## Supplementary material

Data Sheet 1 and Data Sheet 2 Original data for the study of patients in group RAI and non-RAI.

Data Sheet 3 and Data Sheet 4 Code used in R software to development and validation of nomograms.
