# Supplementary material for: Development and validation of nomograms for predicting survival in differentiated thyroid cancer patients with or without radioiodine therapy
Source: Front Oncol. 2023 Mar 9;13:1054594. doi: 10.3389/fonc.2023.1054594 (PMC10034318; doi:10.3389/fonc.2023.1054594)
Supplement: Supplementary file 4 [file DataSheet_4.docx]

setwd("D:/seer")

mydata<-read.csv("DTCNNRAI.csv")

F=c(6:16);for (i in F) mydata[, i] = factor(mydata[, i])

str(mydata)

mydata$Race<-factor(mydata$Race,labels=c('White','Black','Other'))

mydata$Sex <- factor(mydata$Sex,labels=c('male','female'))

mydata$mal <- factor(mydata$mal,labels=c('Papilary','Follicula'))

mydata$Grade <- factor(mydata$Grade,labels=c('I','II','III','IV'))

mydata$T <- factor(mydata$T,labels=c('T1','T2','T3','T4'))

mydata$N <- factor(mydata$N,labels=c('N0','N1'))

mydata$M <- factor(mydata$M,labels=c('M0','M1'))

mydata$Surg <- factor(mydata$Surg,labels=c('NO','Lobectomy','Subtotal or near-total thyroidectomy','Total thyroidectomy'))

mydata$LND <- factor(mydata$LND,labels=c('None or Biopsy','1-3 regional LN','≥4 regional LN','unknown'))

mydata$CHE <- factor(mydata$CHE,labels=c('yes','no/unknown'))

str(mydata)

library(survival)

n<-dim(mydata)[1]

set.seed(9)

ind<-sample(n,0.7*n)

train<- mydata[ind,]

test<- mydata[-ind,]

train$group <- 1

test$group <- 0

mydata2 <- rbind(train,test)

survdiff(Surv(month,status) ~ group,data=mydata2)

mod<-coxph(Surv(month,status)~ Age+size+ELN+PLN+LNR+Race+Sex+mal+Grade+AJCC+T+N+M+Surg+LND+CHE,data=train)

summary(mod)

step(object=mod,direction="both")

mod1<-coxph(Surv(month,status)~Age + size + PLN + Grade + T + N + M + CHE,data=train)

summary(mod1)

library(rms)

fcox1<-cph(Surv(month,status) ~ Age + size + PLN + Grade + T + N + M + CHE,surv=T,x=T, y=T,data=train)

f1 <- cph(Surv(month,status) ~ predict(fcox1,newdata=train), x=T, y=T, surv=T, data=train)

validate(f1, method="boot", B=500, dxy=T)

rcorrcens(Surv(month,status) ~ predict(fcox1, newdata=train), data = train)

fvad1 <-cph(Surv(month,status) ~ predict(fcox1,newdata=test),x=T, y=T,surv=T, data=test)

validate(fvad1, method="boot", B=500, dxy=T)

prcorrcens(Surv(month,status) ~ predict(fcox1, newdata=test), data = test)

mod1<-coxph(Surv(month,status)~ Age + size + PLN + Grade + T + N + M + CHE,data=train)

summary(mod1)

mod2<-coxph(Surv(month,status)~ AJCC,data=train) #AJCC

summary(mod2)

library(rms)

fcox1<-cph(Surv(month,status)~ Age + size + PLN + Grade + T + N + M + CHE,surv=T,x=T, y=T,data=train)

f1 <- cph(Surv(month,status) ~ predict(fcox1,newdata=train), x=T, y=T, surv=T, data=train)

validate(f1, method="boot", B=500, dxy=T)

rcorrcens(Surv(month,status) ~ predict(fcox1, newdata=train), data = train)

fvad1 <-cph(Surv(month,status) ~ predict(fcox1,newdata=test),x=T, y=T,surv=T, data=test)

validate(fvad1, method="boot", B=500, dxy=T)

rcorrcens(Surv(month,status) ~ predict(fcox1, newdata=test), data = test)

fcox2 <- cph(Surv(month,status) ~ AJCC,surv=T,x=T, y=T,data=train)

f2 <- cph(Surv(month,status) ~ predict(fcox2,newdata=train), x=T, y=T, surv=T, data=train)

validate(f2, method="boot", B=500, dxy=T)

rcorrcens(Surv(month,status) ~ predict(fcox2, newdata=train), data = train)

fvad2 <-cph(Surv(month,status) ~ predict(fcox2,newdata=test),x=T, y=T,surv=T, data=test)

validate(fvad2, method="boot", B=500, dxy=T)

rcorrcens(Surv(month,status) ~ predict(fcox2, newdata=test), data = test)

library(rms)

library(foreign)

library(survival)

ddist <- datadist(train)

options(datadist='ddist')

units(train$month) <- "Months"

units(test$month) <- "Months"

fcox<-cph(Surv(month,status)~ Age + size + PLN + Grade + T + N + M + CHE,surv=T,x=T, y=T,data=train)

surv <- Survival(fcox)

nom <- nomogram(fcox, fun=list(function(x) surv(60, x),

function(x) surv(96, x),

function(x) surv(120, x)),

funlabel=c("5-years Survival Probability",

"8-years Survival Probability",

"10-years Survival Probability"),lp=T)

plot(nom,lmgp=0.2,

xfrac=0.7,

naxes=14,

cex.var = 1,

label.every = 2,

cex.axis = 1.1,

col.grid = gray(c(0.95,0.98)))

plot(nom,lmgp=0.2,

xfrac=0.7,

naxes=14,

cex.var = 1,

label.every = 2,

cex.axis = 0.75,

col.grid = gray(c(0.95,0.98)))

library(survivalROC)

nobs<- NROW(train)

cutoff1<- 60

cutoff2<- 96

cutoff3<- 120

coxmod=coxph(Surv(month,status)~ Age + size + PLN + Grade + T + N + M + CHE,data=train)

summary(coxmod)

train$Agepoint <- train$Age* 0.075075

train$sizepoint <- train$size* 0.006284

train$PLNpoint <- train$PLN* 0.032914

Graden<- as.numeric(train$Grade)

train$Gradepoint<- ifelse(Graden==1,0,ifelse(Graden==2, -0.214567,ifelse(Graden==3, 1.249685, 1.938983)))

Tn<- as.numeric(train$T)

train$Tpoint<- ifelse(Tn==1,0,ifelse(Tn==2, 0.471816,ifelse(Tn==3, 0.294272, 1.291013)))

Nn<- as.numeric(train$N)

train$Npoint<- ifelse(Nn==1,0, 1.025743)

Mn<- as.numeric(train$M)

train$Mpoint<- ifelse(Mn==1,0, 0.706610)

CHEn<- as.numeric(train$CHE)

train$CHEpoint<- ifelse(CHEn==1,0, -2.533763)

train$PI<-train$points<-rowSums(train[,c("Agepoint","sizepoint","PLNpoint","Gradepoint","Tpoint","Npoint","Mpoint","CHEpoint")])

summary(train$PI)

SROC= survivalROC(Stime = train$month, status = train$status, marker = train$PI, predict.time =cutoff1, method= "KM" )

cut.op= SROC$cut.values[which.max(SROC$TP-SROC$FP)]

plot(SROC$FP,SROC$TP, type="l", xlim=c(0,1), ylim=c(0,1),

xlab = paste( "FP","\n", "AUC = ",round(SROC$AUC,3)),

ylab = "TP", col="red")

abline(0,1)

legend("bottomright",c("5-year ROC for no-RAI"),col="red",lty=c(1,1))

SROC2= survivalROC(Stime = train$month, status = train$status,marker = train$PI,predict.time =cutoff2, method= "KM" )

cut.op2= SROC2$cut.values[which.max(SROC2$TP-SROC2$FP)]

cut.op2

plot(SROC2$FP,SROC2$TP, type="l", xlim=c(0,1), ylim=c(0,1),

xlab = paste( "FP","\n", "AUC = ",round(SROC2$AUC,3)),

ylab = "TP", col="red")

abline(0,1)

legend("bottomright",c("8-year ROC for no-RAI "),col="red",lty=c(1,1))

SROC3=survivalROC(Stime=train$month,status=train$status,marker=train$PI,predict.time=cutoff3,method="KM")

cut.op3=SROC3$cut.values[which.max(SROC3$TP-SROC3$FP)]

cut.op3

plot(SROC3$FP,SROC3$TP,type="l",xlim=c(0,1),ylim=c(0,1),

xlab = paste( "FP","\n", "AUC = ",round(SROC3$AUC,3)),

ylab = "TP", col="red")

abline(0,1)

legend("bottomright",c("10-year ROC for no-RAI"),col="red",lty=c(1,1))

plot(SROC$FP,SROC$TP, type="l", xlim=c(0,1), ylim=c(0,1),

xlab = paste( "FP"),

ylab = "TP", col="red")

lines(SROC2$FP,SROC2$TP, type="l", xlim=c(0,1), ylim=c(0,1),

xlab = paste( "FP"),

ylab = "TP", col="blue")

lines(SROC3$FP,SROC3$TP, type="l", xlim=c(0,1), ylim=c(0,1),

xlab = paste( "FP"),

ylab = "TP", col="black")

abline(0,1)

legend("bottomright",legend=c("5-year ROC of no-RAI,AUC = 0.891","8-year ROC of no-RAI,AUC = 0.884","10-year ROC of no-RAI,AUC = 0.852"), col=c("red", "blue","black"),lty=c(1,1))

test$Agepoint <- test$Age* 0.075075

test$sizepoint <- test$size* 0.006284

test$PLNpoint <- test$PLN* 0.032914

Graden<- as.numeric(test$Grade)

test$Gradepoint<- ifelse(Graden==1,0,ifelse(Graden==2, -0.214567,ifelse(Graden==3, 1.249685, 1.938983)))

Tn<- as.numeric(test$T)

test$Tpoint<- ifelse(Tn==1,0,ifelse(Tn==2, 0.471816,ifelse(Tn==3, 0.294272, 1.291013)))

Nn<- as.numeric(test$N)

test$Npoint<- ifelse(Nn==1,0, 1.025743)

Mn<- as.numeric(test$M)

test$Mpoint<- ifelse(Mn==1,0, 0.706610)

CHEn<- as.numeric(test$CHE)

test$CHEpoint<- ifelse(CHEn==1,0, -2.533763)

test$PI<-test$points<-rowSums(test[,c("Agepoint","sizepoint","PLNpoint","Gradepoint","Tpoint","Npoint","Mpoint","CHEpoint")])

summary(test$PI)

SROC= survivalROC(Stime = test$month, status = test$status, marker = test$PI, predict.time =cutoff1, method= "KM" )

cut.op= SROC$cut.values[which.max(SROC$TP-SROC$FP)]

plot(SROC$FP,SROC$TP, type="l", xlim=c(0,1), ylim=c(0,1),

xlab = paste( "FP","\n", "AUC = ",round(SROC$AUC,3)),

ylab = "TP", col="red")

abline(0,1)

legend("bottomright",c("5-year ROC for no-RAI"),col="red",lty=c(1,1))

SROC2= survivalROC(Stime = test$month, status = test$status,marker = test$PI,predict.time =cutoff2, method= "KM" )

cut.op2= SROC2$cut.values[which.max(SROC2$TP-SROC2$FP)]

cut.op2

plot(SROC2$FP,SROC2$TP, type="l", xlim=c(0,1), ylim=c(0,1),

xlab = paste( "FP","\n", "AUC = ",round(SROC2$AUC,3)),

ylab = "TP", col="red")

abline(0,1)

legend("bottomright",c("8-year ROC for no-RAI "),col="red",lty=c(1,1))

SROC3=survivalROC(Stime=test$month,status=test$status,marker=test$PI,predict.time=cutoff3,method="KM")

cut.op3=SROC3$cut.values[which.max(SROC3$TP-SROC3$FP)]

cut.op3

plot(SROC3$FP,SROC3$TP,type="l",xlim=c(0,1),ylim=c(0,1),

xlab = paste( "FP","\n", "AUC = ",round(SROC3$AUC,3)),

ylab = "TP", col="red")

abline(0,1)

legend("bottomright",c("10-year ROC for no-RAI"),col="red",lty=c(1,1))

plot(SROC$FP,SROC$TP, type="l", xlim=c(0,1), ylim=c(0,1),

xlab = paste( "FP"),

ylab = "TP", col="red")

lines(SROC2$FP,SROC2$TP, type="l", xlim=c(0,1), ylim=c(0,1),

xlab = paste( "FP"),

ylab = "TP", col="blue")

lines(SROC3$FP,SROC3$TP, type="l", xlim=c(0,1), ylim=c(0,1),

xlab = paste( "FP"),

ylab = "TP", col="black")

abline(0,1)

legend("bottomright",legend=c("5-year ROC of no-RAI,AUC = 0.867","8-year ROC of no-RAI,AUC = 0.896","10-year ROC of no-RAI,AUC = 0.899"), col=c("red", "blue","black"),lty=c(1,1))

library(nricens)

mstd=coxph(Surv(month,status==1) ~ AJCC,x=TRUE,data=train)

mnew=coxph(Surv(month,status==1)~ Age + size + PLN + Grade + T + N + M + CHE,x=TRUE,data=train)

nricens(mdl.std = mstd, mdl.new = mnew, t0 = 60, updown = 'diff',cut = 0.05, niter = 200)

nricens(mdl.std = mstd, mdl.new = mnew, t0 = 96, updown = 'diff',cut = 0.05, niter = 200)

nricens(mdl.std = mstd, mdl.new = mnew, t0 = 120, updown = 'diff',cut = 0.05, niter = 200)

mstd2=coxph(Surv(month,status==1) ~ predict(mstd,newdata=test),x=TRUE,data=test)

mnew2=coxph(Surv(month,status==1) ~ predict(mnew,newdata=test),x=TRUE,data=test)

nricens(mdl.std = mstd2, mdl.new = mnew2, t0 = 60, updown = 'diff',cut = 0.05, niter = 200)

nricens(mdl.std = mstd2, mdl.new = mnew2, t0 = 96, updown = 'diff',cut = 0.05, niter = 200)

nricens(mdl.std = mstd2, mdl.new = mnew2, t0 = 120, updown = 'diff',cut = 0.05, niter = 200)

source("stdca.R")

mstd=coxph(Surv(month,status==1) ~ AJCC,x=TRUE,data=train)

mnew=coxph(Surv(month,status==1)~ Age + size + PLN + Grade + T + N + M + CHE,x=TRUE,data=train)

train$oldfive.years.Survival.Probability=c(1-(summary(survfit(mstd,newdata=train),times=60)$surv))

train$newfive.years.Survival.Probability=c(1-(summary(survfit(mnew,newdata=train),times=60)$surv))

trainevent<-train[train$status==1,]

trainnonevent<-train[train$status==0,]

IDIevent<-trainevent$newfive.years.Survival.Probability-trainevent$oldfive.years.Survival.Probability

IDInonevent<-trainnonevent$newfive.years.Survival.Probability-trainnonevent$oldfive.years.Survival.Probability

mean(IDIevent)

mean(IDInonevent)

IDI=mean(IDIevent)-mean(IDInonevent)

IDI

SEevent = sd(IDIevent)/sqrt(length(IDIevent))

SEnonevnt = sd(IDInonevent)/sqrt(length(IDInonevent))

Z = IDI/sqrt(SEevent*SEevent + SEnonevnt*SEnonevnt )

Z

P= 1- pnorm(Z)

P

train$oldeight.years.Survival.Probability=c(1-(summary(survfit(mstd,newdata=train),times=120)$surv))

train$neweight.years.Survival.Probability=c(1-(summary(survfit(mnew,newdata=train),times=120)$surv))

trainevent<-train[train$status==1,]

trainnonevent<-train[train$status==0,]

IDIevent<-trainevent$neweight.years.Survival.Probability-trainevent$oldeight.years.Survival.Probability

IDInonevent<-trainnonevent$neweight.years.Survival.Probability- trainnonevent$oldeight.years.Survival.Probability

mean(IDIevent)

mean(IDInonevent)

IDI= mean(IDIevent)- mean(IDInonevent)

IDI

SEevent = sd(IDIevent)/sqrt(length(IDIevent))

SEnonevnt = sd(IDInonevent)/sqrt(length(IDInonevent))

Z = IDI/sqrt(SEevent*SEevent + SEnonevnt*SEnonevnt )

Z

P= 1- pnorm(Z)

P

train$oldten.years.Survival.Probability=c(1-(summary(survfit(mstd,newdata=train),times=120)$surv))

train$newten.years.Survival.Probability=c(1-(summary(survfit(mnew,newdata=train),times=120)$surv))

trainevent<-train[train$status==1,]

trainnonevent<-train[train$status==0,]

IDIevent<-trainevent$newten.years.Survival.Probability-trainevent$oldten.years.Survival.Probability

IDInonevent<-trainnonevent$newten.years.Survival.Probability- trainnonevent$oldten.years.Survival.Probability

mean(IDIevent)

mean(IDInonevent)

IDI= mean(IDIevent)- mean(IDInonevent)

IDI

SEevent = sd(IDIevent)/sqrt(length(IDIevent))

SEnonevnt = sd(IDInonevent)/sqrt(length(IDInonevent))

Z = IDI/sqrt(SEevent*SEevent + SEnonevnt*SEnonevnt )

Z

P= 1- pnorm(Z)

P

mstd2=coxph(Surv(month,status==1) ~ predict(mstd,newdata=test),x=TRUE,data=test)

mnew2=coxph(Surv(month,status==1) ~ predict(mnew,newdata=test),x=TRUE,data=test)

test$oldfive.years.Survival.Probability=c(1-(summary(survfit(mstd,newdata=test),times=60)$surv))

test$newfive.years.Survival.Probability=c(1-(summary(survfit(mnew,newdata=test),times=60)$surv))

testevent<-test[test$status==1,]

testnonevent<-test[test$status==0,]

IDIevent<-testevent$newfive.years.Survival.Probability-testevent$oldfive.years.Survival.Probability

IDInonevent<-testnonevent$newfive.years.Survival.Probability-testnonevent$oldfive.years.Survival.Probability

mean(IDIevent)

mean(IDInonevent)

IDI=mean(IDIevent)-mean(IDInonevent)

IDI

SEevent = sd(IDIevent)/sqrt(length(IDIevent))

SEnonevnt = sd(IDInonevent)/sqrt(length(IDInonevent))

Z = IDI/sqrt(SEevent*SEevent + SEnonevnt*SEnonevnt )

Z

P= 1- pnorm(Z)

P

test$oldeight.years.Survival.Probability=c(1-(summary(survfit(mstd,newdata=test),times=96)$surv))

test$neweight.years.Survival.Probability=c(1-(summary(survfit(mnew,newdata=test),times=96)$surv))

testevent<-test[test$status==1,]

testnonevent<-test[test$status==0,]

IDIevent<-testevent$neweight.years.Survival.Probability-testevent$oldeight.years.Survival.Probability

IDInonevent<-testnonevent$neweight.years.Survival.Probability-testnonevent$oldeight.years.Survival.Probability

mean(IDIevent)

mean(IDInonevent)

IDI=mean(IDIevent)-mean(IDInonevent)

IDI

SEevent=sd(IDIevent)/sqrt(length(IDIevent))

SEnonevnt=sd(IDInonevent)/sqrt(length(IDInonevent))

Z=IDI/sqrt(SEevent*SEevent + SEnonevnt*SEnonevnt )

Z

P= 1- pnorm(Z)

P

test$oldten.years.Survival.Probability=c(1-(summary(survfit(mstd2,newdata=test),times=120)$surv))

test$newten.years.Survival.Probability=c(1-(summary(survfit(mnew2,newdata=test),times=120)$surv))

testevent<- test[test$status==1,]

testnonevent<- test[test$status==0,]

IDIevent<-testevent$newten.years.Survival.Probability-testevent$oldten.years.Survival.Probability

IDInonevent<-testnonevent$newten.years.Survival.Probability- testnonevent$oldten.years.Survival.Probability

mean(IDIevent)

mean(IDInonevent)

IDI= mean(IDIevent)- mean(IDInonevent)

IDI

SEevent = sd(IDIevent)/sqrt(length(IDIevent))

SEnonevnt = sd(IDInonevent)/sqrt(length(IDInonevent))

Z = IDI/sqrt(SEevent*SEevent + SEnonevnt*SEnonevnt )

Z

P= 1- pnorm(Z)

P

fcox3<-cph(Surv(month,status)~ Age + size + PLN + Grade + T + N + M + CHE,surv=T,x=T,y=T,time.inc=60,data=train)

cal3 <- calibrate(fcox3, cmethod="KM", method="boot", u=60, m=815, B=500)

plot(cal3)

abline(0,1,lty = 2,col="red")

fcox5 <-cph(Surv(month,status)~ Age + size + PLN + Grade + T + N + M + CHE,surv=T,x=T,y=T,time.inc=96,data=train)

cal5<- calibrate(fcox5, cmethod="KM", method="boot", u=96, m=815, B=500)

plot(cal5)

abline(0,1,lty = 2,col="red")

fcox8<-cph(Surv(month,status)~ Age + size + PLN + Grade + T + N + M + CHE,surv=T,x=T,y=T,time.inc= 120,data=train)

cal8 <- calibrate(fcox8, cmethod="KM", method="boot", u=120, m=815, B=500)

plot(cal8)

abline(0,1,lty = 2,col="red")

fvad3 <- cph(Surv(month,status) ~predict(fcox, newdata=test), x=T, y=T, surv=T, data=test, time.inc=60)

cfvad3 <- calibrate(fvad3, cmethod="KM", method="boot", u=60, m=349, B=500)

plot(cfvad3)

abline(0,1,lty = 2,col="red")

fvad5 <- cph(Surv(month,status) ~predict(fcox, newdata=test), x=T, y=T, surv=T, data=test, time.inc=96)

cfvad5 <- calibrate(fvad5, cmethod="KM", method="boot", u=96, m=349, B=500)

plot(cfvad5)

abline(0,1,lty = 2,col="red")

fvad8 <- cph(Surv(month,status) ~predict(fcox, newdata=test), x=T, y=T, surv=T, data=test, time.inc=120)

cfvad8 <- calibrate(fvad8, cmethod="KM", method="boot", u=120, m=349, B=500)

plot(cfvad8)

abline(0,1,lty = 2,col="red")

mnew=coxph(Surv(month,status)~ Age + size + PLN + Grade + T + N + M + CHE,data=train)

mstd=coxph(Surv(month,status) ~ AJCC,data=train)

train$five.years.Survival.Probabilitynew=c(1-(summary(survfit(mnew,newdata=train),times=60)$surv))

train$five.years.Survival.Probability=c(1-(summary(survfit(mstd,newdata=train),times=60)$surv))

train$eight.years.Survival.Probabilitynew=c(1-(summary(survfit(mnew,newdata=train),times=96)$surv))

train$eight.years.Survival.Probability=c(1-(summary(survfit(mstd,newdata=train),times=96)$surv))

train$ten.years.Survival.Probabilitynew=c(1-(summary(survfit(mnew,newdata=train),times=120)$surv))

train$ten.years.Survival.Probability=c(1-(summary(survfit(mstd,newdata=train),times=120)$surv))

stdca(data=train,outcome="status",ttoutcome="month",timepoint=60,predictors=c("five.years.Survival.Probabilitynew","five.years.Survival.Probability"),xstop=0.6,smooth=TRUE)

stdca(data=train,outcome="status",ttoutcome="month",timepoint=96,predictors=c("eight.years.Survival.Probabilitynew","eight.years.Survival.Probability"),xstop=0.6,smooth=TRUE)

stdca(data=train,outcome="status",ttoutcome="month",timepoint=120,predictors=c("ten.years.Survival.Probabilitynew","ten.years.Survival.Probability"),xstop=0.6,smooth=TRUE)

mnew=coxph(Surv(month,status)~predict(fcox1,newdata=test),data=test)

mstd=coxph(Surv(month,status)~predict(fcox2,newdata=test),data=test)

test$five.years.Survival.Probabilitynew=c(1-(summary(survfit(mnew,newdata=test),times=60)$surv))

test$five.years.Survival.Probability=c(1-(summary(survfit(mstd,newdata=test),times=60)$surv))

test$eight.years.Survival.Probabilitynew=c(1-(summary(survfit(mnew,newdata=test),times=96)$surv))

test$eight.years.Survival.Probability=c(1-(summary(survfit(mstd,newdata=test),times=96)$surv))

test$ten.years.Survival.Probabilitynew=c(1-(summary(survfit(mnew,newdata=test),times=120)$surv))

test$ten.years.Survival.Probability=c(1-(summary(survfit(mstd,newdata=test),times=120)$surv))

stdca(data=test,outcome="status",ttoutcome="month",timepoint=60,predictors=c("five.years.Survival.Probabilitynew","five.years.Survival.Probability"),xstop=0.4,smooth=TRUE)

stdca(data=test,outcome="status",ttoutcome="month",timepoint=96,predictors=c("eight.years.Survival.Probabilitynew","eight.years.Survival.Probability"),xstop=0.3,smooth=TRUE)

stdca(data=test,outcome="status",ttoutcome="month",timepoint=120,predictors=c("ten.years.Survival.Probabilitynew","ten.years.Survival.Probability"),xstop=0.3,smooth=TRUE)

mnew=coxph(Surv(month,status)~ Age + size + PLN + Grade + T + N + M + CHE,data=train)

train$five.years.Survival.Probability=c(1-(summary(survfit(mnew,newdata=train),times=60)$surv))

train$eight.years.Survival.Probability=c(1-(summary(survfit(mnew,newdata=train),times=96)$surv))

train$ten.years.Survival.Probability=c(1-(summary(survfit(mnew,newdata=train),times=120)$surv))

stdca(data=train,outcome="status",ttoutcome="month",timepoint=60,predictors=c("five.years.Survival.Probability"),xstop=0.7,smooth=TRUE)

stdca(data=train,outcome="status",ttoutcome="month",timepoint=96,predictors=c("eight.years.Survival.Probability"),xstop=0.4,smooth=TRUE)

stdca(data=train,outcome="status",ttoutcome="month",timepoint=120,predictors=c("ten.years.Survival.Probability"),xstop=0.4,smooth=TRUE)

mnew=coxph(Surv(month,status)~predict(fcox1,newdata=test),data=test)

test$five.years.Survival.Probability=c(1-(summary(survfit(mnew,newdata=test),times=60)$surv))

test$eight.years.Survival.Probability=c(1-(summary(survfit(mnew,newdata=test),times=96)$surv))

test$ten.years.Survival.Probability=c(1-(summary(survfit(mnew,newdata=test),times=120)$surv))

stdca(data=test,outcome="status",ttoutcome="month",timepoint=60,predictors=c("five.years.Survival.Probability"),xstop=0.4,smooth=TRUE)

stdca(data=test,outcome="status",ttoutcome="month",timepoint=96,predictors=c("eight.years.Survival.Probability"),xstop=0.3,smooth=TRUE)

stdca(data=test,outcome="status",ttoutcome="month",timepoint=120,predictors=c("ten.years.Survival.Probability"),xstop=0.3,smooth=TRUE)

write.csv(test,"DTCNNRAI-test.csv",fileEncoding = 'GBK')

write.csv(train,"DTCNNRAI-train.csv",fileEncoding = 'GBK')
